# Cryptopleurine Targets NF-κB Pathway, Leading to Inhibition of Gene Products Associated with Cell Survival, Proliferation, Invasion, and Angiogenesis

**DOI:** 10.1371/journal.pone.0040355

**Published:** 2012-06-29

**Authors:** Hong Ri Jin, Song Zhu Jin, Xing Fu Cai, Donghao Li, Xue Wu, Ji Xing Nan, Jung Joon Lee, Xuejun Jin

**Affiliations:** 1 Key Laboratory of Natural Resources of Changbai Mountain and Functional Molecules, Yanbian University, Ministry of Education, Yanji, Jilin, China; 2 Department of Immunology and Pathogenic Biology, College of Basic Medicine, Yanbian University, Yanji, Jilin, China; 3 Center for Molecular Cancer Research, Korea Research Institute of Bioscience and Biotechnology, Ochang, Chungbuk, Republic of Korea; Wake Forest University, School of Medicine, United States of America

## Abstract

**Background:**

Cryptopleurine, a phenanthroquinolizidine alkaloid, was known to exhibit anticancer activity; however, the underlying mechanism is poorly understood. Because the nuclear factor-κB (NF-κB) transcription factors control many physiological processes including inflammation, immunity, and development and progression of cancer, we investigated the effects of cryptopleurine on tumor necrosis factor alpha (TNF-α)-induced NF-κB activation pathway and on the expression of NF-κB-regulated gene products associated with many pathophysiological processes.

**Methodology and Principal Finding:**

MDA-MB231, MDA-MB435, MCF-7, HEK293, RAW264.7 and Hep3B cells were used to examine cryptopleurine's effect on the NF-κB activation pathway. Major assays were promoter-reporter gene assay, electrophoretic mobility shift assay (EMSA), *in vitro* immune complex kinase assay, real-time PCR, Western blot analysis, and Matrigel invasion assay. Experiments documenting cell proliferation and apoptosis were analyzed by MTT method and flow cytometry, respectively. The results indicated that cryptopleurine suppressed the NF-κB activation through the inhibition of IκB kinase (IKK) activation, thereby blocking the phosphorylation and degradation of the inhibitor of NF-κB alpha (IκBα) and the nuclear translocation and DNA-binding activity of p65. The suppression of NF-κB by cryptopleurine led to the down-regulation of gene products involved in inflammation, cell survival, proliferation, invasion, and angiogenesis.

**Conclusions and Significance:**

Our results show that cryptopleurine inhibited NF-κB activation pathway, which leads to inhibition of inflammation, proliferation, and invasion, as well as potentiation of apoptosis. Our findings provide a new insight into the molecular mechanisms and a potential application of cryptopleurine for inflammatory diseases as well as certain cancers associated with abnormal NF-κB activation.

## Introduction

The nuclear factor-κB (NF-κB) transcription factors control many physiological processes including inflammation, immunity, apoptosis, and tumor invasion [1], [2], [3]. NF-κB represents a family of related DNA-binding proteins, which in mammals includes five members: NF-κB1 (or p50), NF-κB2 (or p52), RelA (or p65), RelB and c-Rel. In an inactive state, NF-κB is sequestered in the cytoplasm as a heterotrimer consisting of p50, p65, and IκB subunits. On activation, inhibitor of NF-κB alpha (IκBα) undergoes phosphorylation and ubiquitination-dependent degradation leading to p65 nuclear translocation and binding to a specific consensus sequence in the DNA, which results in gene transcription.

It is reported that NF-κB regulates more of than 150 genes, including those involved in immunity and inflammation, anti-apoptosis, cell proliferation, tumorigenesis and the negative feedback of the NF-κB signal [4]. NF-κB regulates major inflammatory cytokines, including interleukin 6 (IL-6), interleukin 8 (IL-8), interleukin-1 beta (IL-1β), many of which are potent activators for NF-κB [5]. Therefore, NF-κB is primarily an inducer of inflammatory cytokines. Its inhibitors could be useful as anti-inflammatory agents [6]. In addition to regulating the expression of genes important for immune and inflammatory responses, NF-κB also controls the transcription of genes that are critical in the early and late stages of aggressive cancers, including cyclooxygenase-2 (COX-2), cyclinD1, apoptosis suppressor proteins such as cellular inhibitor of apoptosis 1 (cIAP1), B-cell lymphoma 2 (Bcl2), TNF-α receptor-associated factor 2 (TRAF2), cellular FLICE inhibitory protein (FLIP), and genes required for invasion and angiogenesis such as inter-cellular adhesion molecule 1 (ICAM-1), matrix metalloproteinase (MMP-9) and vascular endothelial growth factor (VEGF) [7], [8]. Therefore, the NF-κB inhibitors might also be useful as anti-cancer agents.

NF-κB inhibitors including a variety of natural products, chemicals, metals, metabolites, synthetic compounds, peptides, and protein (cellular, viral, bacterial, fungal) can be divided into different groups depended on the target levels of NF-κB signaling: upstream of IκB kinase (IKK), directly at the IKK complex or IκB phosphorylation, ubiquitination, proteasomal degradation of IκB, nuclear translocation of NF-κB, NF-κB-DNA binding, and NF-κB transactivation [9], [10]. To date, a large number of natural compounds have been reported as NF-κB inhibitors and some of these have been further investigated for the application in diseases treatment [11], [12].

Cryptopleurine is a phenanthroquinolizidine alkaloid isolated from the roots of *Boehmeria pannosa* (Urticaceae). Cryptopleurine was shown to have potent antiviral activity against herpes virus and anti-inflammatory [13]. This alkaloid also showed potent anticancer activity against human gastric cancer cells through inhibition of hypoxia-inducible factor-1α [14]. Tumor necrosis factor alpha (TNF-α) is an important proinflammatory factor that acts as a master switch in establishing an intricate link between inflammation and cancer [15]. It contributes to the development of the tissue architecture necessary for tumor growth and metastasis. It also induces other cytokines and angiogenic factors and thus contributes to the increased growth and survival of tumor cells. A wide variety of evidence has pointed to a critical role of TNF-α and the NF-κB pathway on cancer cell survival, proliferation, invasion, and angiogenesis [16], [17]. The results described below showed that cryptopleurine inhibits the TNF-α-induced IκB kinase (IKK) activation, thereby blocking the activation of NF-κB through the inhibition of IκBα phosphorylation and degradation, and p65 nuclear translocation and DNA-binding activity. Furthermore, the suppression of NF-κB by cryptopleurine led to the down-regulation of gene products involved in inflammation (IL-6, IL-8, and IL-1β), cell survival (TRAF2, Bcl2, cIAP1, and FLIP), proliferation (cyclinD1 and COX-2), invasion (ICAM-1 and MMP-9), and angiogenesis (VEGF).

## Results

### Cryptopleurine Inhibits NF-κB Activation by Different Stimuli

**Figure 1:**
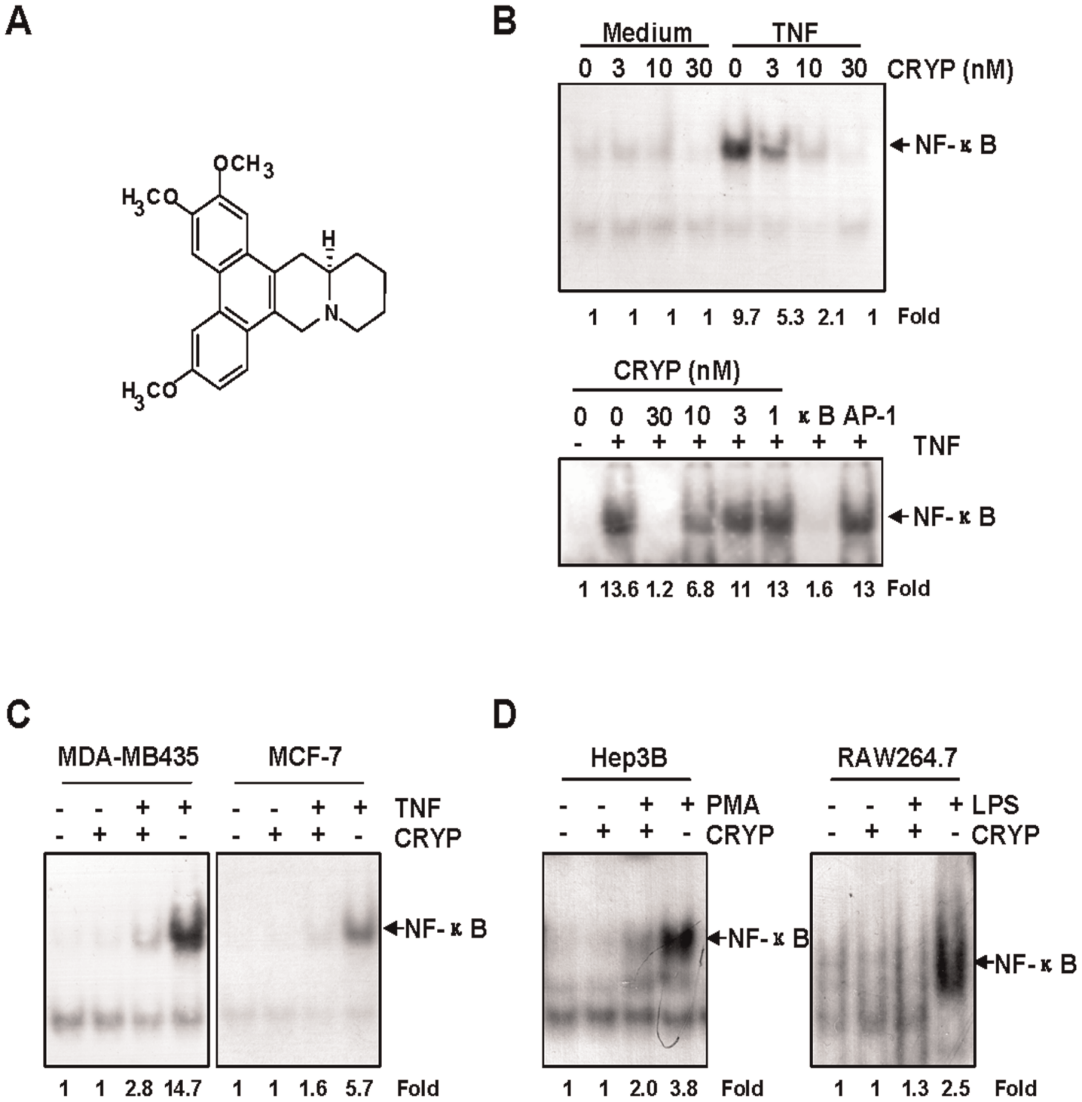
Cryptopleurine inhibits NF-κB activation by different stimuli. (A) Structure of cryptopleurine. (B) MDA-MB231 cells (upper panel) and Hep3B cells (lower panel) were preincubated with indicated concentrations of cryptopleurine for 12 h and then treated with TNF-α (20 ng/ml) for 15 min. Nuclear extracts were then prepared and assayed for NF-κB activation by EMSA. In lane AP-1, a 100-fold excess of unlabeled AP-1 consensus oligonucleotide was added to the reaction mixture. In lane κB, a 100-fold excess of unlabeled κB consensus oligonucleotide was added to the reaction mixture. The arrow indicates the location of the DNA-NF-κB complex. (C) MDA-MB435, and MCF-7 cells were incubated with 30 nM cryptopleurine for 12 h and then incubated with 20 ng/ml TNF-α for 15 min. Nuclear extracts were then prepared and assayed for NF-κB activation by EMSA. (D) Hep3B, and RAW264.7 cells were preincubated with 30 nM cryptopleurine for 12 h and then treated with 25 ng/ml PMA or 1 μg/ml LPS for 90 min. Nuclear extracts were then prepared and assayed for NF-κB activation by EMSA.

In an effort to unravel the molecular mechanism of cryptopleurine (Fig. 1A), we investgated its effects on TNF-α-induced NF-κB activation pathway. In MDA-MB231 cells and Hep3B cells, cryptopleurine suppressed TNF-α-induced NF-κB activation in a dose-dependent manner, and this inhibition was observed at a concentration even as low as 30 nM (Fig. 1B upper panel and lower panel). Since AP-1 transcription factor is also regulated by TNF-α, we examined whether the compound show specificity on the regulation of NF-κB but not AP-1. Electrophoretic mobility shift assay (EMSA) using excess NF-κB- or AP-1-consensus oligonucleotide showed that cryptopleurine is not likely to regulate TNF-α induced AP-1 activity (Fig. 1B lower panel). Similar inhibitory effect of cryptopleurine on NF-κB activation by TNF-α was observed in MDA-MD435 and MCF-7 cells (Fig. 1C). Since PMA and LPS are potent activators of NF-κB [18], we compared the effect of cryptopleurine on the activation of NF-κB induced by TNF-α, PMA, and LPS. EMSA showed that cryptopleurine suppressed the NF-κB activation induced by all these agents (Fig. 1D), suggesting that cryptopleurine may target one or more common step to the stimuli in the NF-κB activation pathway.

### Cryptopleurine Inhibits TNF-α-Induced IκBα Phosphorylation and Degradation

**Figure 2:**
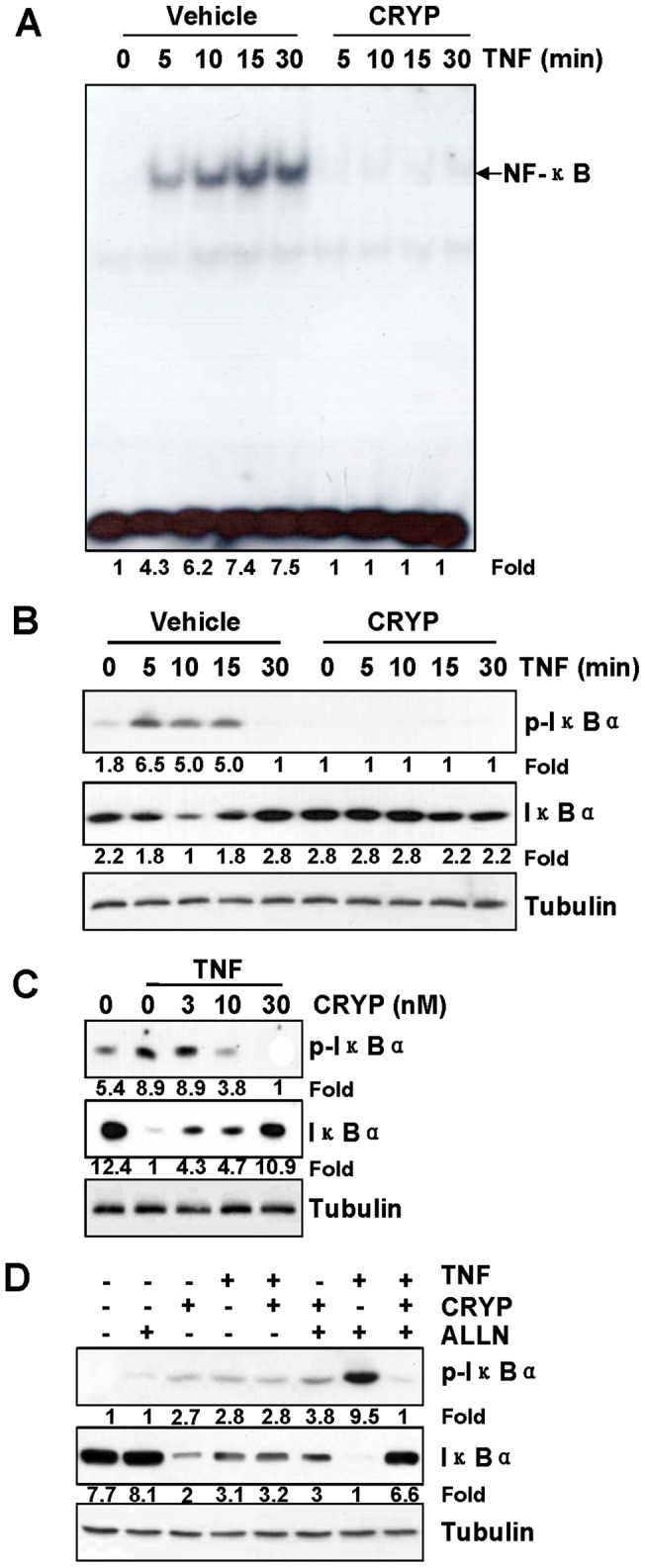
Effect of cryptopleurine on the TNF-α-induced phosphorylation and degradation of IκBα. (A) MDA-MB231 cells were incubated with 30 nM cryptopleurine for 12 h and then incubated with 20 ng/ml TNF-α for the indicated times. Nuclear extracts were then prepared and assayed for NF-κB activation by EMSA. (B) MDA-MB231 cells were incubated with 30 nM cryptopleurine for 12 h and then incubated with 20 ng/ml TNF-α for the indicated times. Cytoplasmic extracts were analyzed by Western blot using indicated antibodies for p-IκBα, IκBα, and tubulin. (C) MDA-MB231 cells were preincubated with indicated concentrations of cryptopleurine for 12 h and then treated with TNF-α (20 ng/ml) for 15 min. Cytoplasmic extracts were analyzed by Western blot using indicated antibodies for p-IκBα, IκBα, and tubulin. (D) MDA-MB231 cells were preincubated with 30 nM cryptopleurine for 12 h, incubated with 50 μg/ml ALLN for 30 min, and then treated with 20 ng/ml TNF-α for 15 min. Cytoplasmic extracts were analyzed by Western blot using indicated antibodies for p-IκBα, IκBα, and tubulin.

To determine how cryptopleurine inhibits TNF-α-induced NF-κB activation, we exposed the MDA-MB231 cells to cryptopleurine for 12 h and then treated them with TNF-α for different periods. We then prepared nuclear extracts and cytoplasmic extracts and analyzed them for NF-κB activation by EMSA and for phosphorylation and degradation of IκBα by Western blot. The results showed that TNF-α induced NF-κB activation in a time-dependent manner and that the earliest activation occurred within 5 min after TNF-α addition (Fig. 2A, left four lanes). However, pretreatment of 30 nM of cryptopleurine blocked TNF-α-induced NF-κB activation (Fig. 2A, right four lanes). TNF-α induced phosphorylation and degradation of IκBα were also occurred as quickly as 5 min (Fig. 2B, left five lanes). However, cryptopleurine potently inhibited the TNF-α-induced phosphorylation and degradation of IκBα (Fig. 2B, right five lanes), and this inhibitory effect was also observed in a dose-dependent manner (Fig. 2C). Since a rapid degradation of phosphorylated IκBα occurred in 5 min, we used a proteasome inhibitor ALLN to block degradation of IκBα. Western blot analysis showed that TNF-α induced IκBα phosphorylation and degradation, whereas cryptopleurine suppressed both events (Fig. 2D). All of the results consistently support the idea that cryptopleurine inhibits TNF-α-induced activation of NF-κB through inhibition of phosphorylation and degradation of IκBα.

### Cryptopleurine Inhibits TNF-α-Induced Activation of IκBα Kinase, p65 Phosphorylation, and p65 Nuclear Translocation

**Figure 3:**
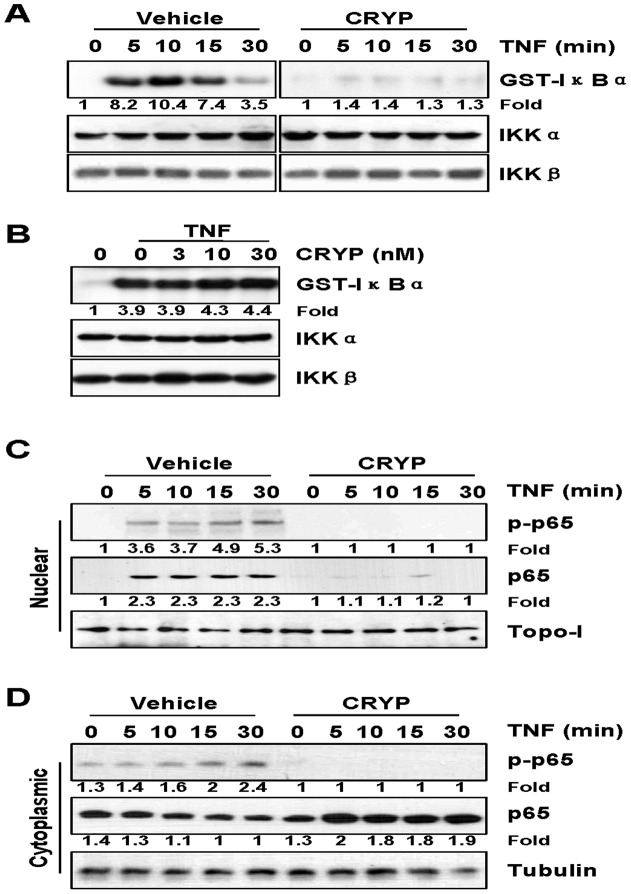
Effect of cryptopleurine on the TNF-α-induced activation of IκBα kinase, p65 phosphorylation, and p65 nuclear translocation. (A) HEK293 cells transfected with IKKα or IKKβ stimulated with TNF-α for the indicated times in the presence or absence of 30 nM cryptopleurine. Whole-cell extracts were immunoprecipitated with antibody for IKKα or IKKβ, and *In vitro* kinase assays were performed with GST-IκBα and [γ-^32^p]ATP. To show the equal amount of immunocomplex in each reaction, the bottom gels represent IKKα or IKKβ detected with Western blot. (B) HEK293 cells transfected with IKKα or IKKβ stimulated with TNF-α for 15 min, and whole-cell extracts were immunoprecipitated with antibody for IKKα. Indicated concentrations of cryptopleurine was added to the immunoprecipitated IKK complex, incubated for 30 min, and analyzed by an immune complex kinase assay. MDA-MB231 cells were incubated with 30 nM cryptopleurine for 12 h and then incubated with 20 ng/ml TNF-α for the indicated times. Nuclear extracts (C) and cytoplasmic extracts (D) were analyzed by Western blot using indicated antibodies for Ser536 phosphorylation in p65, p65, tubulin, and Topo-I.

Since IKK complex acts as a convergence point for a variety of upstream signalings and plays a critical role in phosphorylation and degradation of IκBα proteins [19], we examined whether cryptopleurine inhibits TNF-α-induced activation of IKK with *in vitro* kinase assay. TNF-α induced IKK activation in a time-dependent manner (Fig. 3A, left five lanes) and cryptopleurine (30 nM) inhibited this activation (Fig. 3A, right five lanes). Neither TNF-α nor cryptopleurine had any effect on the expression of IKK proteins. To evaluate whether cryptopleurine directly inhibited IKK activity, we immunoprecipitated IKK complex from 293 cells transfected with IKKα or IKKβ after TNF-α stimulation, and then *in vitro* kinase assays were conducted in the presence of various concentrations of cryptopleurine. Results from the immune complex kinase assay showed that cryptopleurine does not directly affect the IKK activity (Fig. 3B) but rather modulates the activation of IKK induced by TNF-α. Since TNF-α also induces the phosphorylation of p65, which is required for its transcriptional activity [20], we examined whether cryptopleurine affects TNF-α-induced phosphorylation of p65 at Ser536. Either phosphorylated form of p65 or p65 were not detectable in the nuclear fraction in the presence of cryptopleurine, while TNF-α alone did induce the phosphorylation and translocation of p65 (Fig. 3C, top and middle panel). Similar results were obtained with cytoplasmic p65 phosphorylation (Fig. 3D, top and middle panel). TNF-α induced phosphorylation of p65 time-dependently, however, cryptopleurine blocked TNF-α induced phosphorylation of the cytoplasmic of p65 while the p65 level was not significantly changed (Fig. 3D, top and middle panel). These results indicated that the inhibition of IKK activation by cryptopleurine would be associated with the blocking of TNF-α-induced phosphorylation and nuclear translocation of p65.

### Cryptopleurine Inhibits TNF-α-Induced NF-κB-Dependent Reporter Gene Expression

**Figure 4:**
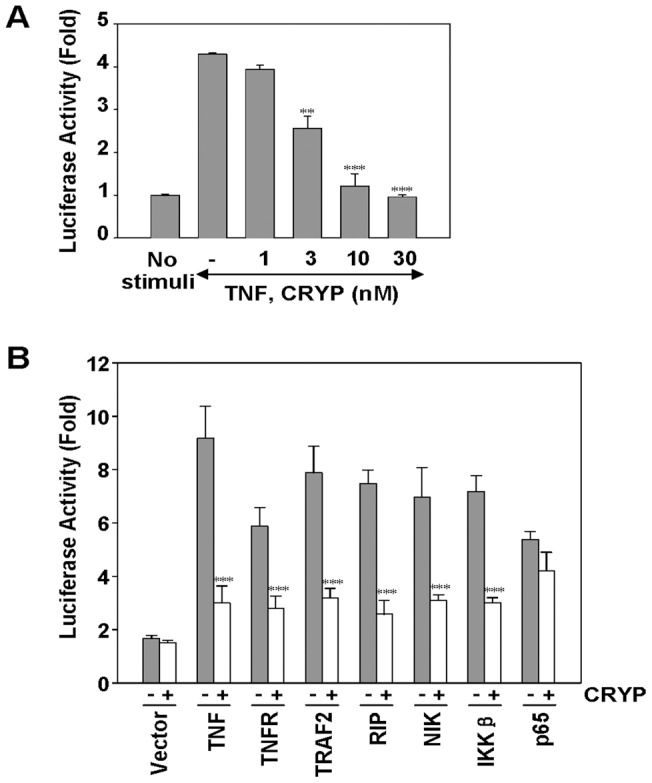
Effect of cryptopleurine on the TNF-α-induced NF-κB-dependent reporter gene expression. (A) MDA-MB231 cells were transiently transfected with a NF-κB reporter construct pNF-κB-Luc for 24 h. After transfection, cells were incubated with indicated concentrations of cryptopleurine for 12 h and then treated with 20 ng/ml TNF-α for an additional 12 h. The lysates of MDA-MB231 cells were subject to the measurement of dual luciferase activity. Data represented as mean ± standard deviation of three independent experiments. **p<0.01, ***p<0.001, significantly different when compared with TNF-α-stimulated normal cells. (B) MDA-MB231 cells were transiently transfected with a NF-κB reporter construct pNF-κB-Luc alone or with plasmids expressing the indicated proteins. After transfection, cells were incubated with 30 nM cryptopleurine for 12 h and then incubated with the relevant plasmid for an additional 12 h. TNF-α-treated cells were incubated with 30 nM cryptopleurine for 12 h and then treated with 20 ng/ml TNF-α for an additional 12 h. The lysates of MDA-MB231 cells were subject to the measurement of dual luciferase activity. Data represented as mean ± standard deviation of three independent experiments. ***p<0.001, significant with respect to control.

Next, we investigated whether cryptopleurine modulates NF-κB-dependent reporter gene expression. After cells were transiently transfected with the NF-κB-regulated luciferase reporter vector, the cells were further incubated with TNF-α in the presence of various concentration of cryptopleurine. We found that TNF-α-induced NF-κB reporter activity was substantially suppressed by cryptopleurine in a dose-dependent manner (Fig. 4A). Since TNF-α-induced NF-κB activation pathway, which is mediated through the sequential interaction of TNFR and TRADD, TRAF2, NIK, and IKK, results in the phosphorylation and degradation of IκBα to realease NF-κB [21], we further examine the effects of cryptopleurine on the TNF-α-induced NF-κB activation pathway in the MDA-MB231 cells transfected with the NF-κB-regulated luciferase reporter gene and plasmids expressing TNFR1, TRAF2, RIP, NIK, IKK, or p65. The cells were then treated with cryptopleurine and monitored for NF-κB-dependent reporter gene expression. We found that cryptopleurine suppressed the NF-κB activation induced by all plasmids but p65 (Fig. 4B). This result suggest that NF-κB-dependent transcription induced by overexpression of RelA/p65 was not likely affected by cryptopleurine significantly, indicating that cryptopleurine could not influence the transactivation activity of RelA/p65 subunit.

### Cryptopleurine Inhibits the Expression of TNF-α-Induced NF-κB-Dependent Inflammatory Cytokines, Antiapoptotic, and Proliferation Genes

**Figure 5:**
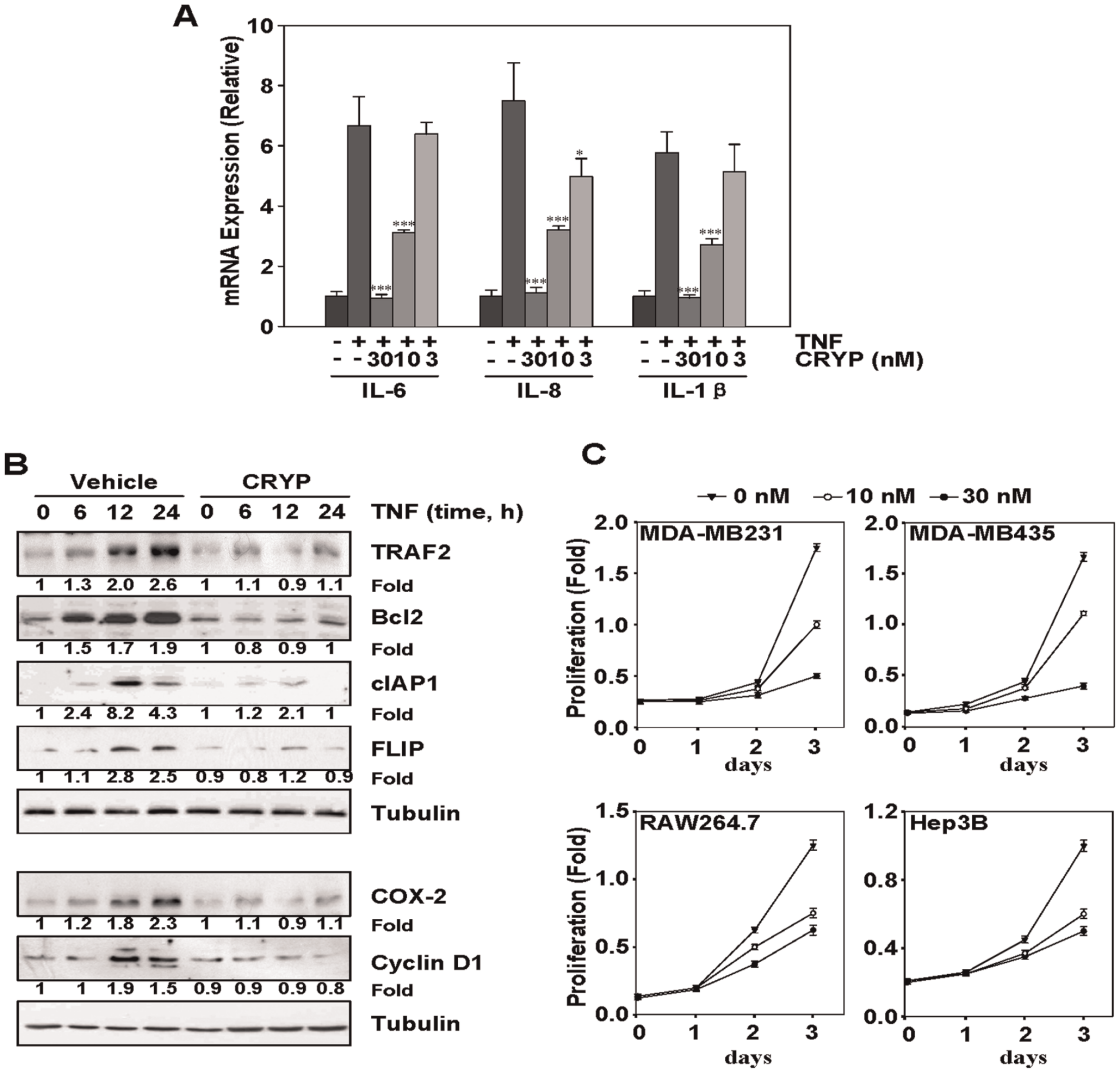
Effect of cryptopleurine on the TNF-α-induced NF-κB-dependent inflammatory cytokines, antiapoptotic, and proliferation genes expression. (A) MDA-MB231 cells were preincubated with indicated concentrations of cryptopleurine for 12 h and then treated with TNF-α (20 ng/ml) for an additional 12 h. RNA was isolated from cells, reverse-transcribed, and analyzed by real-time PCR for IL-6, IL-8, and IL-1β. Data represented as mean ± standard deviation of three independent experiments. *p<0.05, ***p<0.001, significantly different when compared with TNF-α-stimulated normal cells. (B) MDA-MB231 cells were incubated with 30 nM cryptopleurine for 12 h and then incubated with 20 ng/ml TNF-α for the indicated times. Whole cell extracts were analyzed by Western blot using indicated antibodies for TRAF2, Bcl2, cIAP1, FLIP, COX-2, cyclinD1, and tubulin. (C) MDA-MB231, MDA-MB435, RAW264.7, and Hep3B cells were plated in triplicate, treated with 0, 10 or 30 nM cryptopleurine, and subjected to MTT assay on days 1, 2, 3 to analyze cell proliferation. Absorbance was measured at 570 nm.

Next, we investigated the effect of cryptopleurine on TNF-α-induced expression of IL-6, IL-8, and IL-1β in MDA-MB231 cell. After MDA-MB231 cells were stimulated with 20 ng/ml TNF-α for 12 h in the presence or absence of various concentrations of cryptopleurine, the expression of IL-6, IL-8, and IL-1β was measured by quantitative real-time PCR. Cryptopleurine significantly suppressed the TNF-α-induced expression of IL-6, IL-8, and IL-1β in a dose-dependent manner (Fig. 5A). Because NF-κB regulates the expression of the antiapoptotic proteins such as, TRAF2 [22], Bcl_2_ [23], cIAP1 [24], FLIP [25], we examined whether cryptopleurine can modulate the expression of these antiapoptotic genes products induced by TNF-α. MDA-MB231 cells were preincubated with cryptopleurine for 12 h and subsequently stimulated with TNF-α for different periods, and then the TRAF2, Bcl_2_, cIAP1, and FLIP expression were analyzed by Western blot. TNF-α significantly induced the expression of antiapoptotic proteins in a time-dependent manner, whereas cryptopleurine markedly suppressed TNF-α-induced expression of all these proteins (Fig. 5B, top five panels). We also analyzed the effect of cryptopleurine on the TNF-α-induced expression of cyclinD1 and COX-2, both of which are associated with tumor cells proliferation [26], [27]. We observed that TNF-α induced the expression of cyclinD1 and COX-2 and that cryptopleurine significantly suppressed their expression (Fig. 5B, bottom three panels). Because cryptopleurine suppressed gene expression involved in proliferation, we examined whether cryptopleurine alone can modulate the proliferation of various tumor cell types. Cryptopleurine by itself marginally suppressed the proliferation of human breast cancer cells (MDA-MB231 and MDA-MB435), and human hepatic carcinoma cells (Hep3B), and mouse macrophage cells (RAW264.7) at concentration of 30 nM for three days treatment (Fig. 5C).

### Cryptopleurine Potentiates TNF-α-Induced Apoptosis

**Figure 6:**
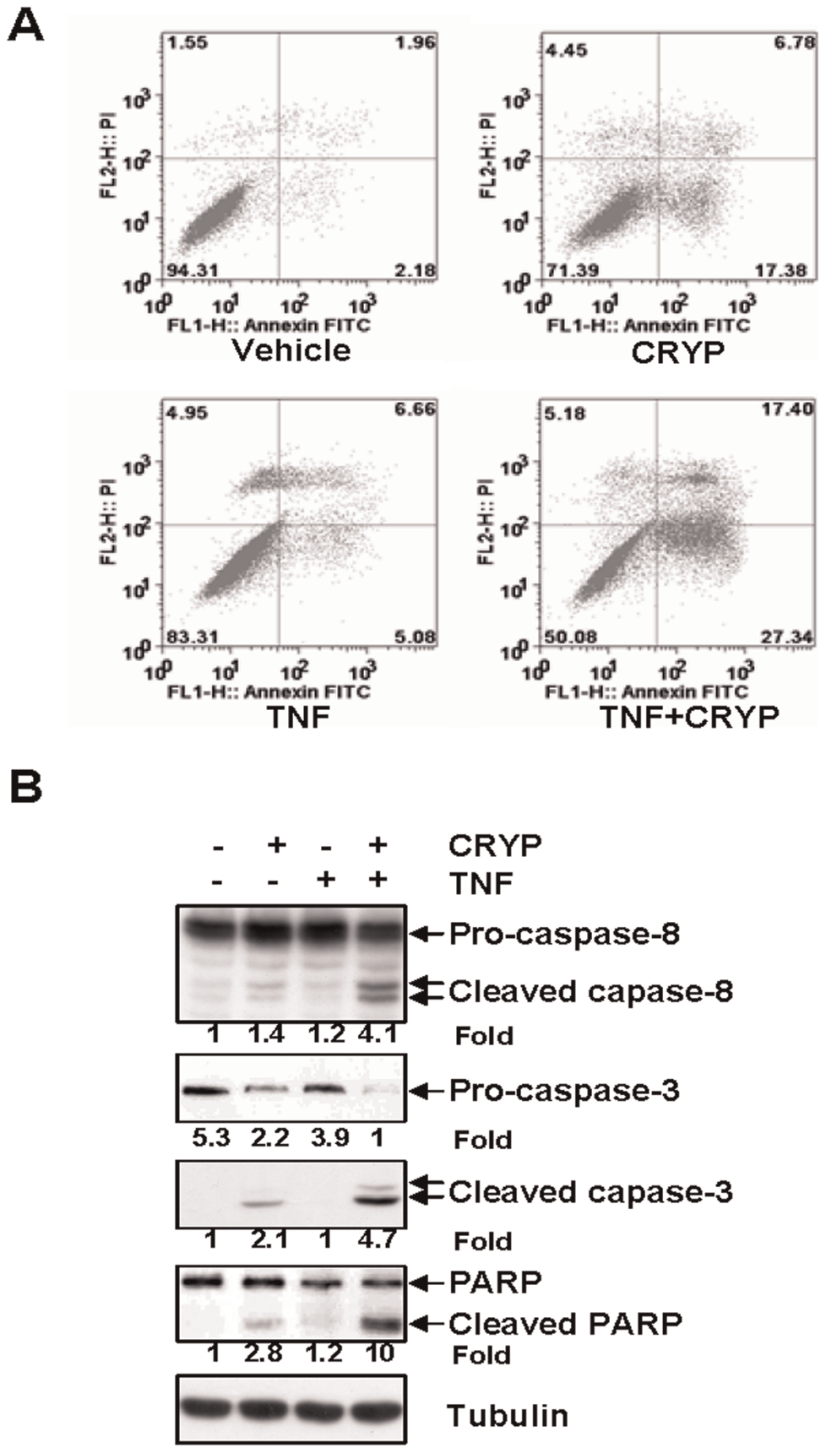
Effect of cryptopleurin on the TNF-α-induced apoptosis. (A) MDA-MB231 cells were pretreated with 30 nM cryptopleurine for 12 h and then incubated with 20 ng/ml TNF-α for 24 h, and subsequently stained with Annexin V-FITC and propidium iodide, followed by analysis using a flow cytometer. Representative plots of one set of triplicate experiments. Early apoptotic cell (Annexin-V^+^ and PI) were displayed in the lower right quadrant and late apoptotic cells (Annexin-V^+^ and PI^+^) were shown in the upper right quadrant. (B) MDA-MB231 cells were pretreated with 30 nM cryptopleurine for 12 h and then incubated with 20 ng/ml TNF-α for 24 h. Whole cell extracts were analyzed by Western blot using indicated antibodies for cleaved capase-8, cleaved capase-3, cleaved PARP, and tubulin.

Because TNF-α-induced expression of antiapoptotic genes was downregulated by cryptopleurine, so we examined whether cryptopleurine enhances apoptosis induced by TNF-α. Cryptopleurine potentiated TNF-α-induced apoptosis, as assessed by Annexin V/PI double staining. As shown in Fig. 6A, combined treatment resulted in a significant increased the Annexin V-positive cell population (44.74%), whereas no treatment (4.14%), treatment with TNF-α alone (11.74%) or cryptopleurine alone (24.16%) has a little influence on the cell apoptosis. Since caspases are a group of cysteine proteases critical for apoptosis of eukaryotic cells [28], we investigated whether cryptopleurine affects TNF-α-induced activation of caspase-8 and caspase-3. TNF-α alone negatively and cryptopleurine alone significantly affected the activation of caspase-8 or caspase-3, whereas cotreatment with TNF-α and cryptopleurine potentiated their activation, as indicated by the presence of cleaved caspases (Fig. 6B, top three panels). We also used the PARP cleavage assay to detect TNF-α-induced apoptosis. Again, cryptopleurine potentiated the effect of TNF-α-induced PARP cleavage, although cryptopleurine alone also induced PARP cleavage (Fig. 6B, bottom second panel). These results showed that cryptopleurine enhances the apoptotic effects of TNF-α.

### Cryptopleurine Inhibits TNF-α-Induced Invasion Activity

**Figure 7:**
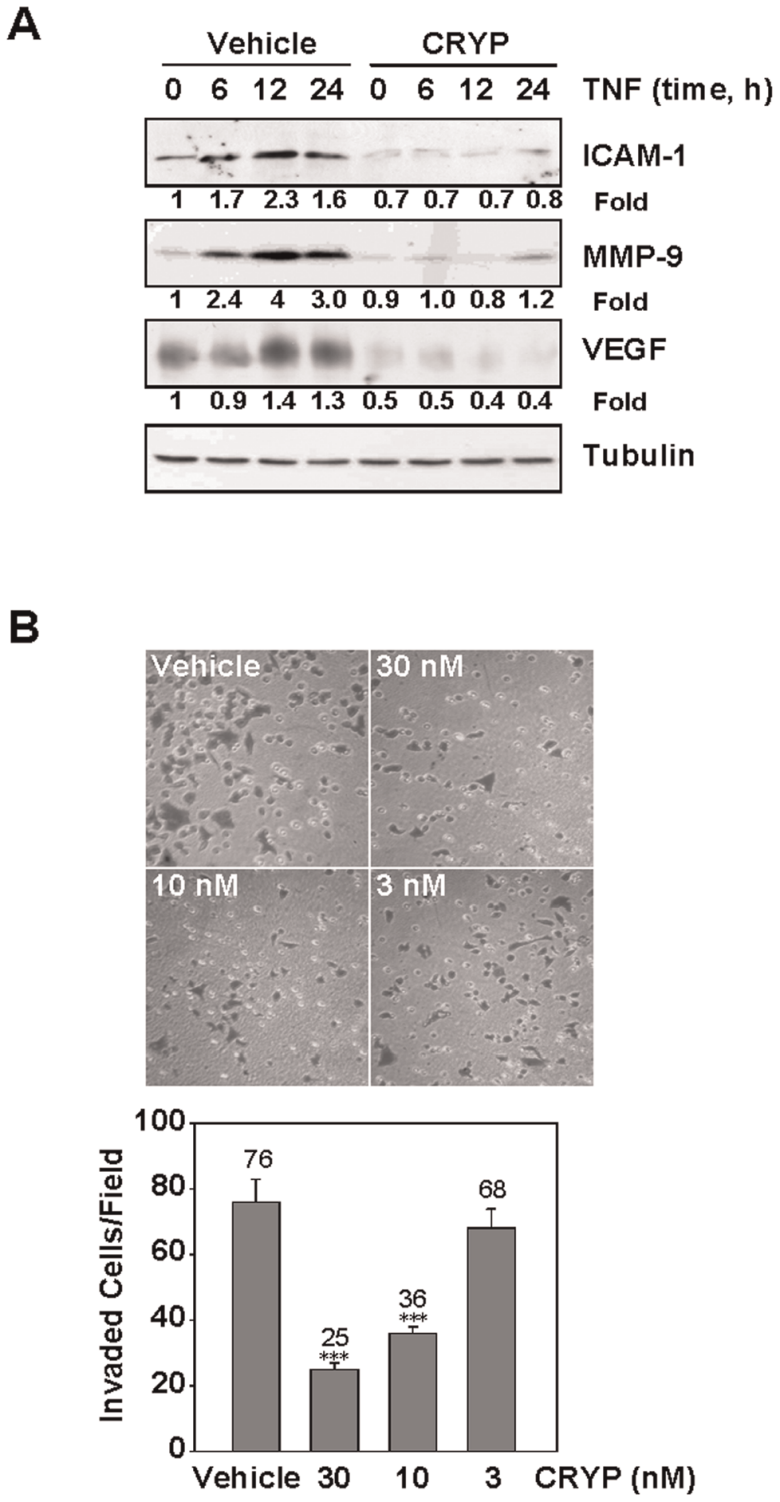
Effect of cryptopleurine on the TNF-α-induced invasion activity. (A) MDA-MB231 cells were incubated with 30 nM cryptopleurine for 12 h and then incubated with 20 ng/ml TNF-α for the indicated times. Whole cell extracts were analyzed by Western blot using indicated antibodies for ICAM-1, MMP-9, VEGF, and tubulin. (B) MDA-MB231 cells invaded through the pores in the Matrigel-coated filters were fixed, stained and counted in five random field visualized by microscopy (100×). Data represented as mean ± standard deviation of three independent experiments. ***p<0.001, significant with respect to control.

The expression of ICAM-1, MMP-9 and VEGF, which are involved in tumor cells invasion and metastasis, is known to be regulated by NF-κB [29], [30], [31]. We therefore examined whether cryptopleurine can suppress the expression of these proteins. TNF-α treatment induced the expression of ICAM-1, MMP-9 and VEGF in time-dependent manner, whereas cryptopleurine inhibited their expression (Fig. 7A). Reduced expression of ICAM-1, MMP-9 and VEGF might be responsible for diminished invasion of tumor cells in cryptopleurine treatment. Therefore, whether cryptopleurine modulates invasion activity was examined *in vitro* with a Matrigel invasion assay. MDA-MB231 cells were seeded in the top chamber of a Matrigel invasion chamber and were incubated with various concentrations of cryptopleurine for 24 h. The result could account for the anti-invasive activity of cryptopleurine (Fig. 7B).

## Discussion

The present study reports for the first time that cryptopleurine inhibits NF-κB. Plant of the *Boehmeria pannosa* is a rich source of phenanthroquinolizidine alkaloid. Extracts containing phenanthroquinolizidine alkaloid, cryptopleurine have been used in traditional medicine such as anti-viral, anti-fungal, anti-bacterial, anti-amoebic, and anti-tumor activities [14], [32]. Despite of its various pharmacological activities, its molecular mechanism has not been sufficiently explained. In this study, we identified cryptopleurine as a potent inhibitor of stimuli-induced NF-κB activation and investigated further how this compound suppressed NF-κB activation.

Our results show that cryptopleurine significantly blocked the NF-κB activation induced by various stimuli including TNF-α, PMA, and LPS, suggesting that cryptopleurine interferes with one or more common steps during NF-κB activation rather than with one single event. Treatment of cells with cryptopleurine potently inhibits the IκB phosphorylation and degradation induced by TNF-α. The IκBα phosphorylation at ser-32 and ser-36 by IKK complex and subsequently degradation by the ubiquitin/proteasome pathway is an essential step for NF-κB activation, it is important to determine whether cryptopleurine directly inhibits IKK complex activation or the activity of upstream kinase activity. Our results show that cryptopleurine did not directly inhibit the activity of IKK but blocked the activation of this kinase. It is well-known that RelA/p65 is a critical transactivation subunit of NF-κB [33], [34]. The transactivation activity of RelA/p65 subunit is regulated by posttranslational modifications such as phosphorylation and acetylation [35]. It was demonstrated that the phosphorylation status of RelA/p65 determines whether it associates with CREB-binding protein/p300, which is a critical regulator of NF-κB [36]. Evidence have been presented that IKK is also critical for the phosphorylation of p65 induced by cytokines such as TNF-α [5]. These observations led us to further observe if cryptopleurine affect p65 phosphorylation induced by TNF-α. As expected, our results show that cryptopleurine significantly inhibits TNF-α-induced RelA/p65 phosphorylation and nuclear translocation. However, NF-κB activity via overexpression of p65 was not likely to be inhibited by cryptopleurine as shown with celastrol, which inhibited IKK activity via C179 modification in the activation loop of IKK [37]. This further indicated that cryptopleurine do not affect transcriptional activity of p65.

Increasing evidences suggested NF-κB pathway plays a crucial role in a link between inflammation and cancer, and demonstrated an essential role for NF-κB in various cancers and inflammatory diseases [7], [38]. Studies have shown that anti-apoptotic activity of NF-κB involves the inhibition of TNF-α-induced apoptosis through induction of a variety of anti-apoptotic [39], [40]. Our results show that cryptopleurine inhibits the TNF-α-induced expression of antiapoptotic proteins such as TRAF2, Bcl2, cIAP1, and FLIP, all of which are known to be regulated by NF-κB. Annexin V staining also showed that TNF-α-induced apoptosis was enhanced by cryptopleurine. In addition, cryptopleurine affects TNF-α-induced activation of caspase-8, caspase-3, and PARP cleavage, although cryptopleurine alone resulted in minimal effect to their activation. Particulary, cryptopleurine had a great effect on TNF-α-induced poly(ADP-ribose) polymerase cleavage, indicating that the apoptotic effects of TNF-α are enhanced by cryptopleurine. Positive effect of cryptopleurine alone on the activation of caspases-8, -3, and PARP cleavage is likely closely associated with the effect of cryptopleurine on the antiproliferation and apoptotsis. It is therefore possible that the activity of cryptopleurine potentiates TNF-α-induced caspases activity and cancer cell death, at least in part, via its NF-κB inhibition. NF-κB also controls the genes expression which important for cell cycle and proliferation [7]. CyclinD1 exercises powerful control over the mechanisms that regulate the mitotic cell cycle. COX-2 is a major mediator of cellular proliferation and survival. The inhibition of the expression of these two genes by cryptopleurine is likely to be associated with inhibition of the proliferation of tumor cells as well. MMP-9 and ICAM-1 have been shown to be expressed in response to NF-κB activation, and are known to be major mediators of tumor cell invasion [41]. Consequently, regulation of these proteins by cryptopleurine was accompanied with suppression of tumor cell invasion as demonstrated in invasion assay. Furthermore, our results indicate that the expression of VEGF, one of the major mediators of angiogenesis, is suppressed by cryptopleurine. Taken together with previous report accounted potent inhibition of hypoxia-inducible factor-1 accumulation in AGS human gastric cancer cells [14], it is highly likely that cryptopleurine can suppress cancer cells survival, proliferation, and metastasis.

In summary, our results suggest that cryptopleurine exhibits antiproliferative, proapoptotic, anti-invasive, antiangiogenic, and anti-inflammatory effects through the suppression of NF-κB and NF-κB-regulated gene products. Our results imply that cryptopleurine could be an interesting lead compound for the modulation of inflammatory diseases as well as certain cancers in which inhibition of NF-κB-activity may be desirable.

## Materials and Methods

### Cell Culture and Reagents

MDA-MB231, MDA-MB435, HEK293, RAW264.7, and Hep3B cells were grown in DMEM with penicillin (100 units/ml)-streptomycin (100 units/ml) (Invitrogen, Carlsbad, CA, USA) and 10% heat-inactivated fetal bovine serum (Hyclone, Logan, UT, USA). MCF-7 cells were maintained in RPMI medium supplemented as above. All cells were purchased from American Type Culture Collection (ATCC, Manassas, VA, USA). TNF-α was obtained from R&D Systems (Minneapolis, MN, USA), phorbol 12-myristate 13-acetate (PMA) and lipopolysaccharide (LPS) from Sigma Chemical Co. N-acetyl-leucyl-leucyl-norleucinal (ALLN) was purchased from Calbiochem (SanDiego, CA, USA). Cryptopleurine was isolated from *Boehmeria pannosa* as described previously [14] and its structures is shown in Fig. 1A. The purity of cryptopleurine was over 98% in HPLC analysis.

### Plasmids, Transfections, and Luciferase Reporter Assay

A pNF-κB-Luc plasmid for NF-κB luciferase reporter assay was obtained from Strategene (LaJolla, CA, USA). Expression vectors were obtained as followed: Flag-IKKα, Flag-IKKβ, and Flag-p65 (M. Karin, University of California San Diego), Myc-NIK and Flag-TNFR (M. Jung, Georgetown University), HA-TRAF2 and HA-RIP were developed in our laboratory. Transfections were performed as described previously [42]. NF-κB-dependent luciferase activity was measured using the Dual Luciferase Reporter Assay system.

### Apoptosis Assays

Annexin V-staining was performed using Annexin V-FITC apoptosis detection kit (BD Biosciences, CA, USA) following the instructions of the manufacturer. Briefly, after incubation, cells were harvested, washed with PBS (pH 7.4), centrifuged, and stained with Annexin V-FITC and 2 μg/ml propidium iodide in binding buffer (10 mM Hepes, pH 7.4, 140 mM NaCl, 2.5 mM CaCl_2_) for 15 min at 37°C in the dark. The samples were analyzed by flow cytometry using a FACScan flow cytometer. The CellQuest software was used to analyze the data (Becton-Dickinson).

### Preparation of Nuclear Extracts and Electrophoretic Mobility Shift Assay (EMSA)

Electrophoretic mobility shift assay was performed as described previously [37]. In brief, prior to stimulation, cells were preincubated with the indicated concentrations of cryptopleurine at 37°C for 12 h. In following, cells were stimulated, harvested by centrifugation, washed twice with cold phosphate-buffered saline, and then nuclear extracts were prepared using NE-PER reagent (Pierce, Rockford, IL, USA), according to the manufacturer's instructions. A double-stranded oligonucleotide for NF-κB (Promega, Madison, WI, USA) was end-labeled with [γ-^32^P] ATP and purified with a G-25 spin column (Boehringer Mannheim, Mannheim, Germany). Nuclear extracts were incubated for 20 min at room temperature with a gel shift binding buffer [5% glycerol, 1 mM MgCl_2_, 0.5 mM EDTA, 0.5 mM DTT, 50 mM NaCl, 10 mM Tris-HCl, pH 7.5, 50 μg/ml poly(dI-dC) poly(dI-dC)] and ^32^P-labeled oligonucleotide. The DNA-protein complex formed was separated on 4% native polyacrylamide gel, and the gel was transferred to Whatman 3 MM paper, dried, and exposed to X-ray film. The specificity of binding was examined by competition with an excess of unlabelled oligonucleotide for NF-κB or AP-1 (Promega, Madison, WI, USA).

### Western Blot Analysis

Whole-cell extracts were obtained by lysing cells in ice-cold lysis buffer (50 mM Tris-HCl, pH 7.5, 1% Nonidet P-40, 1 mM EDTA, 1 mM phenylmethyl sulfonylfluoride) supplemented with the protease inhibitor cocktail (BD Biosciences, San Diego, CA, USA). In certain experiments, the nuclear extracts were prepared using NE-PER reagent. An aliquot of protein extracts were used to determine protein concentration by the Bradford method. Fifty μg of whole-cell extracts or thirty μg of nuclear extract protein per lane was separated by SDS-polyacrylamide gels and followed by transferring to a polyvinylidene difluoride membrane (Millipore, Bedford, MA, USA). The membrane was blocked with 5% skim milk, and then incubated with the corresponding antibody. Antibodies for IκBα, phosphor (Ser32)-specific IκBα, p65, phosphor (Ser536)-specific p65, PARP, caspase-3, caspase-8, and cIAP1 were purchased from Cell Signaling Technology (Beverly, MA, USA). Antibodies for COX-2, ICAM-1, MMP-9, VEGF, cyclinD1, Bcl2, FLIP, IKKα, and IKKβ were obtained from Santa Cruz Biotechnology (Santa Cruz, CA, USA). Antibody for TRAF2 was from R&D system (Minneapolis, MN, USA). Antibody for a-tubulin was from Sigma (St. Louis, MO, USA). After binding of an appropriate secondary antibody coupled to horseradish peroxidase, proteins were visualized by enhanced chemiluminescence according to the manufacturer's instructions (Amersham Pharmacia Biotec, Buckinghamshire, UK).

### Kinase Assays

Human HEK293 cells grown in 100mm plates were transfected with expression vectors for IKKα or IKKβ and incubated for 24 h, and stimulated with TNF-α (20 ng/ml) for 15 min, and then washed three times with ice-cold PBS containing 1 mM Na_3_VO_4_ and 5 mM EDTA. Cell lysates prepared in lysis buffer (20 mM Tris-HCl, 0.5 M NaCl, 0.25% Triton X-100, 1 mM EDTA, 1 mM EGTA, 10 mM β-glycerophosphate, 10 mM NaF, 10 mM 4-nitrophenylphosphate, 300 mM Na_3_VO_4_, 1 mM benzamidine, 2 mM PMSF, 10 mg/ml aprotinin, 1 mg/ml leupeptin, 1 mg/ml pepstatin, and 1 mM DTT) were incubated with IKKα or IKKβ antibody on ice for 2 h. Protein A- or protein G-conjugated agarose beads were then added and incubated for additional 2 h at 4°C. Kinase assays were performed by incubating the immune complexes in kinase reaction buffer (20 mM HEPES, pH 7.7, 2 mM MgCl_2_, 10 mM ATP, 10 mM β-glycerophosphate, 10 mM NaF, 300 mM Na_3_VO_4_, 1 mM benzamidine, 2 mM PMSF, 10 mg/ml aprotinin, 1 mg/ml leupeptin, 1 mg/ml pepstatin, and 1 mM DTT) with 5 μCi of [γ-^32^P] ATP and bacterially expressed GST-IκBα in a reaction volume of 20 μl for 30 min at 30°C. Samples were analyzed by 12.5% SDS-PAGE, autoradiography, and Western blot.

### Real-Time PCR

Real-time PCR was performed as previously described [43]. In brief, RNA was isolated from cells using RNeasy Mini kits according to the manufacturer's instructions (Qiagen, CA, USA). Complementary DNA was synthesized from 1 μg of total RNA in a 20 μl reverse transcription reaction mixture according to the manufacturer's protocol (TaKaRa Bio, Kyoto, Japan). The following primer pairs were used for real-time PCR amplification: human interleukin-6 (IL-6), 5′-GAACTCCTTCTCCACAAGCGCCTT -3′ and 5′-CAAAAGACCAGTGATGATTTTCACCAGG-3′; human interleukin-8 (IL-8), 5′-TCTGCAGCTCTGTGTGAAGG-3′ and 5′-ACTTCTCCACAACCCTCTG-3′; human interleukin-1 beta (IL-1β), 5′-ATGGCAGAAGTACCTAAGCTCGC-3′ and 5′-ACACAAATTGCATGGTGAAGTCAGTT-3′. GAPDH, 5′-ACCAGGTGGTCTCCTCT-3′ and 5′-TGCTGTAGCCAAATTCGTTG-3′. GAPDH was used as the housekeeping gene control. The quantitative real-time PCR was carried out using power SYBR green (BIO-RAD, CA, USA). Reactions were performed in triplicate according to the manufacturer's protocol.

### *In vitro* Invasion Assays

The ability of cells to invade through Matrigel-coated filters was determined using a modified 24-well Boyden chamber (Corning Costar, Cambridge, MA, USA; 8 μm pore size). MDA-MB231 cells were seeded at a density of 5×10^4^ cells in 100 μl DMEM containing 10% FBS in the upper compartment of transwell. To determine the effect of cryptopleurine, various concentrations of cryptopleurine were added to the lower or upper compartment of transwell. After incubation for 24 h at 37°C in 5% CO_2_, the cells that not penetrated the filter were completely wiped out with a cotton swabs, and the cells that had migrated to the lower surface of the filter were fixed, stained, and counted in 5 randomly selected microscopic fields (100×) per filter.

### Statistical Analysis

All values are expressed as mean ± SD. A comparison of the results was performed with one-way ANOVA and Tukey's multiple comparison tests (Graphpad Software, Inc, San Diego, CA, USA). Statistically significant differences between groups were defined as *p*-values less than 0.05.
